# A Lunchtime Walk in Nature Enhances Restoration of Autonomic Control during Night-Time Sleep: Results from a Preliminary Study

**DOI:** 10.3390/ijerph13030280

**Published:** 2016-03-03

**Authors:** Valerie F. Gladwell, Pekka Kuoppa, Mika P. Tarvainen, Mike Rogerson

**Affiliations:** 1 Centre for Sports and Exercise Science, School of Biological Sciences, University of Essex, Wivenhoe Park, Colchester, Essex CO4 3SQ, UK; mrogerp@essex.ac.uk; 2 Department of Applied Physics, University of Eastern Finland, P.O. Box 1627, Kuopio 70211, Finland; pekka.kuoppa@uef.fi (P.K.); mika.tarvainen@uef.fi (M.P.T.); 3 Department of Clinical Physiology and Nuclear Medicine, Kuopio University Hospital, P.O. Box 100, Kuopio 70029, Finland

**Keywords:** green exercise, nature, heart rate variability, vagal activity, autonomic function, walking, recovery

## Abstract

Walking within nature (Green Exercise) has been shown to immediately enhance mental well-being but less is known about the impact on physiology and longer lasting effects. Heart rate variability (HRV) gives an indication of autonomic control of the heart, in particular vagal activity, with reduced HRV identified as a risk factor for cardiovascular disease. Night-time HRV allows vagal activity to be assessed whilst minimizing confounding influences of physical and mental activity. The aim of this study was to investigate whether a lunchtime walk in nature increases night-time HRV. Participants (*n* = 13) attended on two occasions to walk a 1.8 km route through a built or a natural environment. Pace was similar between the two walks. HRV was measured during sleep using a RR interval sensor (eMotion sensor) and was assessed at 1–2 h after participants noted that they had fallen asleep. Markers for vagal activity were significantly greater after the walk in nature compared to the built walk. Lunchtime walks in nature-based environments may provide a greater restorative effect as shown by vagal activity than equivalent built walks. Nature walks may improve essential recovery during night-time sleep, potentially enhancing physiological health.

## 1. Introduction

Exposure to nature has wide ranging positive effects on health, particularly mental wellbeing in comparison to indoor environments [1] or synthetic or built environments [2]. Exercising within nature (termed Green Exercise) is psychologically restorative, immediately enhancing mood and self-esteem [3,4,5]. A handful of studies have investigated the potential of Green Exercise to promote immediate physiologically restorative effects [6,7,8,9,10,11] with fewer assessing longer-lasting effects [2]. 

Compared to equivalent non-Green Exercise conditions (either viewing scenes of built environments on a screen whilst exercising on an ergometer or performing physical activity in an indoor or “built” outdoor environment), Green Exercise participation promotes post-exercise decreases in heart rate (HR) [8,9,10], systolic (SBP) [6,8,10] and diastolic blood pressure (DBP) [6,8,10]. Light intensity walking in a natural environment enhances physiological markers indicative of recovery from stress [7], possibly due to blunted responses in catecholamines and cortisol [9,10,11]. Interestingly, alterations in autonomic nervous system control (ANS) (indirectly measured using heart rate variability (HRV)), have also been reported. HRV is a well-established non-invasive tool giving an indication of the changes in vagal and sympathetic control of the heart, by providing a measurement of inter-beat differences in HR that takes into account both the parasympathetic and sympathetic contributions to the sino-atrial node regulation of HR [12].

The ANS plays an important role in the maintenance of homeostasis and in modulating responses to stressors [10]. Green Exercise has been shown to enhance cardiac vagal activity (parasympathetic activity). To date most of the effects of Green Exercise have been measured either during or immediately after exercise; which may mask benefits of green due to the effects of exercise [13], particularly when exploring the ANS which will be directly affected by the exercise. When measures are taken one hour or more following Green Exercise, even in a controlled environment, additional physical or psychological and sensory inputs that are not related to the Green Exercise (or control activity) may alter physiology markers. The longer-term physiologically restorative properties of Green Exercise remain unexplored.

Sleep is a behavior that promotes bodily restoration and is characterized by circadian variation in metabolic, endocrine and autonomic systems. HRV monitoring during sleep has identified a nocturnal zenith in cardiac vagal activity coincident with peak melatonin and a nadir in circulating cortisol concentrations [14]. Augmenting vagal activity during sleep could enhance restoration and sleep quality. 

There is evidence to suggest a relationship between exercise and sleep quality [15,16,17], but to date, the majority of research has been reliant on self-report methodologies. Two hours of forest walking increased duration of sleep and self-reported sleep quality [18]. Considered together with the outlined previous research findings, which forward that nature environments promote physiological restoration, it seems possible that Green Exercise participation might maximize sleep-related physiological restoration associated with time spent in nature. Despite the outlined current knowledge linking physiological restoration, environmental exposure, physical activity and sleep; research is yet to examine the impact of performing physical activity within different environments on autonomic function during sleep. The current study tested the hypothesis that lunchtime walking in nature increases nocturnal cardiac parasympathetic activity compared with a built walk. 

## 2. Method

### 2.1. Participants

Ethical approval from the University ethics committee was granted (10/BS/130/VG) and *n* = 13 (6 females) participants (mean ± SD: age 39.4 ± 13.9 years; stature 1.69 ± 0.1 m; mass 70.1 ± 15.8 kg) were recruited from University support staff and those in the community living close to the University. All were independent from the research group conducting the study. Due to a technical problem (*i.e.*, sensor fell off during night-time) data were not recorded for one participant (1 female) and this data-set was therefore not included in the analyses. 

Participants were free from symptoms of disease and were not using medication that would affect ANS or the cardiovascular system. Participants were also given written and verbal instructions, and these were checked prior to the commencement of data collection regarding physical activity, food and fluid intake. They were asked to minimize their physical activity and to use a car or public transport to get to and from work (the cost of this was reimbursed). They were also asked to refrain from undertaking any structured exercise for 48 h prior to testing and on day of testing. No alcohol was allowed for 24 h prior to testing and on day of test. Further, it was asked that participants refrained from caffeine on the day of their tests. A diary was kept to ensure that participants were reminded of their diet, physical activity and transport behaviors 48 h prior to and for the rest of the day on their first test day and participants were instructed to use their diary to ensure that activities and food were consistent for the second test.

### 2.2. Study Design

A within-subject, randomized counterbalanced design was used, where participants selected an envelope containing the phrase “built” or “green” on the first walk and completed this type of walk first, with the other environment on the second visit. This resulted in seven participants completing the built walk first (four males) and six completing the Green walk (four males). There were no significant differences in mass by trial order. However, age was significantly different between the groups (Green walk first: M = 55.5 ± 10.1 years; Built walk first: M = 33.6 ± 6.1 years; *p* < 0.0001).

On each of the two occasions, participants completed the walks, starting and finishing at the laboratory. Occasions differed only by the environmental route along which the walk was completed (Green, Built). Two walking routes were identified on the University of Essex campus, UK which were 1.8 km (measured by GPS watch (Forerunner 201, Garmin, Schaffhusen, Switzerland) and a trundle wheel). In the Green condition, the walk route was through grassland, wooded areas and a small lake. Only towards the end of the walk were buildings highly visible. The Built walk ran between student dwellings, roadways, shops and other campus buildings. The Built walk had more people present than the Green walk as would be expected. All walks were completed during May–August and walks did not take place in rain. In these instances visits were rescheduled. Occasions were separated by a minimum of 7 days. Visits were at the same time of day to eliminate any effect of circadian rhythm on the dependent variables and were on the same week day (where possible) to ensure a similar daily routine was followed. Participants had been instructed to wear appropriate clothing and footwear for partaking in moderate exercise outdoors both on tarmac and grassy fields or tracks. 

### 2.3. Measures and Procedures

On arrival, participants completed an informed consent, a physical activity readiness questionnaire (PAR-Q) and the International Physical Activity Questionnaire (I-PAQ), on which participants report their physical activity levels of the last 7 days. With the participant in the seated position, the experimenter measured participants’ BP, using an Omron MXR3 (Omron Healthcare UK Ltd., Milton Keynes, UK). This was performed to ensure consistency in resting cardiovascular measures between testing days. In order to obtain intensity of exercise a HR monitor chest strap (S610i Polar Electro Oy, Kempele, Finland) was fitted, with data collected set to average HR every 5 s. Data were downloaded after the walks were completed using Polar HR monitor software (Polar Electro Oy, Kempele, Finland). Participants then completed a walk along the route applicable to the given condition. During the walk participants were accompanied by a research assistant to show them the route but participants set the pace of walking. Participants were instructed to walk at a tempo that was comfortable to them and represented their normal walking pace. In order to ensure engagement with the environment participants were asked to keep talking to a minimum and that they should “take in the view around whilst walking”. Ratings of perceived exertion (RPE) accounting for whole walk were obtained at completion of each condition using Borg’s (6–20) scale [19,20].

Upon completion of the walks participants were provided with an eMotion sensor (Mega Electronics Ltd., Kuopio, Finland) in order to collect RR interval data overnight following the walks. The eMotion sensor is small and lightweight, and easy to fit, making it suitable for collection of data in a non-laboratory environment. Participants were instructed via demonstration and both written and verbal guidelines of how and where to attach the sensor and how to switch it on. The position of the sensor was in a modified Lead II configuration (as recommended by manufacture and also by Iber *et al*. [21]). Sampling frequency was 1000 Hz which is greater than recommended for sleep studies [21]. Participants were asked to attach the sensor approximately 30 min prior to going to bed. They were asked to note down when the sensor was attached, when they went to bed and the following morning the approximate time they fell asleep. Participants were also asked to record any awakenings in the night to enable data to be analyzed during sleeping periods. Participants returned the HR monitor the following morning after overnight recording. RR interval data were downloaded into eMotion LAB software before further analysis (Mega Electronics Ltd., Kuopio, Finland).

### 2.4. HRV Analysis

Before analysis data were examined for ectopic beats. If any ectopic beats were found then they were removed from the HRV analysis in order to normalize the data. Very low frequency trend components (frequencies below 0.04 Hz) were removed using a smoothness priors approach [22].

In order for data to be able to be interpreted correctly, HRV analysis should be undertaken during slow wave sleep [23] and this typically occurs in the first period of sleep, usually within the first four hours [24]. Recordings of RR intervals that are averaged over several hours do not necessarily give an accurate representation of ANS modulation [12]. There is a need for RR interval data stationarity *i.e*., that if modulations of heart rate are too variable then this hinders a clear interpretation of the data [12]. A single data-analyst blinded to the condition identified stationary data, by selecting three 10 min segments that were within 60–120 min (mean 93 ± 23.6 min) after the participant noted in a diary they had fallen asleep. Usually, the 10 min segments were consecutive to each other but occasionally, larger gaps between data segments were required due to non-stationarity of data or movement artefacts. All HRV analyses were performed using Kubios HRV software (University of Eastern Finland, Kuopio, Finland) [25]. The results were averaged over the three 10 min segments. 

In the time-domain, mean RR interval and number of beats *per* minute (HR) were calculated. Standard deviation of RR interval (SDNN) was chosen to reflect overall HRV (sympathetic and parasympathetic activity). To reflect parasympathetic system activity root mean squared of successive differences (rMSSD) and Poincare plot (SD1) were used [12]. 

### 2.5. Statistical Analysis

Version 21 of SPSS (IBM Corp. Released 2012. IBM SPSS Statistics for Windows, Version 21.0. IBM Corp.: Armonk, NY, USA) was used for all statistical analysis. Differences in all outcome variables according to condition were analyzed by comparing means (SD) and calculating mean difference (95% CI). To determine potential covariates, pre-walk values for resting HR and BP, as well as total Walk time, exercise-HR, change in HR above resting during and RPE were analyzed using repeated measures *t*-test. Repeated measures ANOVA (*F*, *p*, partial eta squared, (η_p_^2^)) were used to describe the magnitude of between-condition differences in overnight changes in HRV. To ensure there was no influence of order effects, secondary analysis was undertaken which included order (coded as a dummy variable 0/1) as a covariate in repeated measures ANOVA. The unadjusted means, mean difference (95% CI) and results of repeated measures ANOVA (*F*, *p*, η_p_^2^) are shown in Table 1 alongside results for repeated measures ANOVA adjusted for condition order. Effect sizes (η_p_^2^) were interpreted as follows: 0.01 small, 0.06 medium, 0.14 large [26]. 

## 3. Results

### 3.1. Resting Cardiovascular Measures

There were no significant differences in cardiovascular measures at rest prior to the two walks for: HR (Green walk: M = 67.3 ± 13.1 bpm; Built walk: M = 65.7 ± 11.7 bpm; *p* = 0.49); SBP (Green walk: M = 118.8 ± 15.0 mmHg; Built walk: M = 119.7 ± 13.0 mmHg; *p* = 0.75); DBP (Green walk: M = 75.3 ± 11.2 mmHg; Built walk: 76.7 ± 8.3 mmHg; *p* = 0.33). 

### 3.2. The Walks and Immediate Effects

The temperature on the days when the walks took place was between 16.1 and 21.8 °C (M = 17.5 °C). Duration of the walks was similar (Green walk: M = 17.3 ± 8.6 min; Built walk: M = 16.9 ± 6.3 min; *p* = 0.13). Neither HR during walking, change in HR above resting nor RPE at completion of walking differed significantly between conditions (Table 2). 

### 3.3. Overnight Effects of the Walks

Sleep duration was 20 min longer following the Built walk than after Green walk but this small difference was not significant (Table 1). HRV indicators (SDNN, rMSSD and SD1) during sleep following Green Walk were all significantly higher than following Built walk (Table 1). Notably, these positive impacts of the Green walk on HRV during sleep were found independent of the order in which participants took the two walks. There was no difference in overnight RR interval between conditions. 

## 4. Discussion

The current study is the first to explore alterations in the ANS during the early part of a night sleep following a bout of Green Exercise at lunchtime. The current study was exploratory and extends the current literature by examining the longer-lasting health enhancing effects of Green Exercise. It is the first study to compare a short walk in a Green environment to walking in a built environment on night-time HRV measures. Perhaps surprisingly, the hypothesis was supported; compared to a built walk, a short Green walk at lunchtime resulted in significantly augmented parasympathetic activity during sleep. That is, both the overall HRV measure (SDNN) and the more specific parasympathetic measures (rMSSD and SD1) were significantly greater in night-time recordings following the Green walk. This may suggest that individuals following a Green walk experience greater restoration during this period of sleep. However, the current study did not extend duration of sleep following a Green walk. This finding is in contrast to previous work that identified greater duration of sleep after a 2 h forest walk in comparison to a night with no walking during the previous day [18]. The previous study compared walking in contrast to no walking and the exercise itself may have caused the enhanced sleep duration. The short walk in the present study may have had a smaller effect than a longer walk *i.e*., it has a smaller stimulus. The intention of the current study, however, was to explore whether walking during lunchtime in a workplace setting altered night-time vagal activity. A two hour walk would not be plausible in this setting. 

As human beings have lived in the natural environment for most of their existence (over 5 million years), it is likely that the physiology of humans is best suited for natural environments and these are likely to promote relaxation and restoration. Previously reported physiological measures obtained during time spent either in natural or simulated environments with or without exercise have provided evidence to support this argument [6,10,27,28]. The results of the current study are consistent with previously reported findings that adrenaline, noradrenaline and BP remain reduced in the evening following a daytime walk in a forest [8]. As the parasympathetic nervous system is dominant in restoration [10,27,28,29], the current findings support the notion that compared to equivalent exercise in other environmental settings, Green Exercise promotes physiological restoration and health. Immediate and short-term similar physiological benefits of Green Exercise have been previously reported [8], but this is the first study to suggest that effects on ANS control, and in particular on parasympathetic activity, are longer lasting and might promote physiological restoration during sleep [30]. Enhanced vagal activity at night-time is necessary for restoration and is essential for health. High vagal activity during sleep is part of the normal circadian variation and is observed in healthy individuals, decreases with age [31] and is reduced in cardiovascular disease [32]. The potential capacity of Green Exercise for elevating nocturnal vagal activity will be useful to explore further. Interestingly, in the current study mean RR interval was not different between the two walks. This may be in part that RR interval was already high during sleep (having a ceiling effect). Additionally, there will be an increase in vagal neuron excitability occurred followed by a rebound inhibition of these vagal neurons by inspiratory drive, leading to augmented HRV values, but with an unchanged mean RR interval (*i.e*., there is greater fluctuation around the mean (as seen by SDNN) but the mean does not change). 

To date studies that have explored physiological responses following Green Exercise, even in a controlled environment, have found it difficult to compare changes in physiology especially when collecting data greater than one hour following exercise. It is likely that the physiological changes that may occur as a result of being exposed to different environments are masked by the physiological effects of the exercise itself [13], and this is particularly important when exploring the ANS via HRV. Measuring during sleep might be advantageous as individuals are likely to be experiencing minimal physical disturbances (inactivity of voluntary muscles) and psychological inputs from external sources, as at this time consciousness is in a reduced or absent state, and sensory activity is limited. It may, however, be useful in future to explore HRV in the one hour following the walks within a controlled environment to allow comparisons to be made to overnight recordings.

A strength of the current study was its cross-over, within subject design, with participants acting as their own control by completing both walks (Green and Built). This ensured that the between subject variability prevalent in the HRV data did not confound the statistical analysis, thus facilitating greater statistical power for the given sample size. Additionally, there were no differences in cardiovascular measures between testing days confirming that all participants were starting with a similar baseline on both occasions. We carefully controlled for duration and intensity of the walk. This was confirmed by mean HR during walks, change in HR from baseline and RPE during both Green and Built walks were comparable. Temperature during both Green and Built walks was also similar. It is therefore unsurprising that there was minimal effect of which order the walks were completed in. 

The duration of the walks in the current study (approximately 17 min) was comparatively shorter than the 2 h used in a previous study of this kind [18]. This indicates that as well as longer walks during leisure time, short walks during lunchtimes, perhaps at workplaces, may promote restorative benefits beyond the immediate future. This suggests that a shorter walk, which fits into a working day, may induce longer lasting physiological benefits. Previous work has shown that as little as five minutes can alter short-term changes in mood [3]. Further investigation is required to identify the optimum dose (duration and intensity) of Green Exercise for enhanced physiological outcomes during sleep.

HRV data was collected for a period 60–120 min from sleep onset (as noted in diary). Although this time period was selected to reflect the minimal sensory input and muscle activity, it is possible that the data analyzed included some data representative of rapid eye movement sleep. However, this is unlikely due to minimal artifacts in ECG, stationarity and almost identical RR intervals for both walks.

There are several further limitations to the current study. The first is the small sample size. However, the *p* values and effect sizes suggest that there appears to be some interesting alterations in HRV which should be explored further. Although the participants completed the walks in a random order, those that completed the Green walk first were significantly older as a group than those who undertook the Built walk. It may be that this may have influenced the results but no order effect for physiological measures was identified. Another potential limitation is that the research assistant consciously or unconsciously may have encouraged more positivity during the Green walk. This is true of any walks where participants are guided. In the design of the current study it was considered whether participants could walk without a guide but this was not deemed plausible as the walks that were used had many changes in direction and without a guide the participant may have become lost or walked an alternative route. Additionally, in future it would be useful to compare HRV recordings following the walk with overnight recordings. However, HRV needs to be collected in a controlled environment, and it is possible that immediately following walks, any effects of environmental settings on HRV may still be masked by effects of the recently performed exercise. That is, environmental influences on HRV may only become measureable when the masking influences of exercise have subsided.

The aim of this preliminary study was to investigate ANS activity during normal habitual sleep and record physiological data with minimal disturbance to the participant *i.e*., within the home setting. As participants remained in their regular and home settings following the walk, it ensured good ecological validity of the demonstrated findings. Allowing the participant, however, to undertake normal habitual activities within their normal settings may potentially affect the physiological outcomes several hours later, including during sleep. To try to eliminate this participants were asked to complete a diary and were asked to keep the second day as similar to the first day as much possible. In the current exploratory study, the alternative of asking participants to stay within a controlled environment was not plausible. Controlling the environment following the walk, including the sleep environment, should be considered for future work. 

Additionally, other relevant physiological measures including actigraphy and/or EEG should be examined in future work. Actigraphy has been used previously [18] before and after forest walking and can give an indication of sleep duration and activity during sleep. Combining HRV and actigraphy may therefore be very useful and give a more accurate indication of sleep time. If a more detailed sleep analysis is required by obtaining the EEG, the participant would need to be placed in a controlled sleeping environment, but this may affect their habitual sleep patterns, influencing HRV measures. Both actigraphy and EEG will allow a more thorough investigation of whether the sleep becomes more restorative by altering the duration of slow wave sleep, rather than altering the duration of sleep for the whole night *i.e*., the quality of sleep increases. This might have important implications to enhance the restorative part of sleep or help those that have sleep disorders. Most sleep disorders have modifications in the physiology of sleep, which manifests itself in signs and symptoms of sleep loss and quality, which are shown to be independent risk factors for cardiovascular morbidity and mortality [33,34]. Therefore ANS cardiovascular control could be a potential physio-pathological link between sleep disorders and their physiological consequences [30]. If Green Exercise could modify ANS control during sleep, this would have important implications for health and wellbeing. Further work is also warranted to explore duration and intensity of the walks and the dose of nature itself, as well of the time of day that individuals perform such walks. 

## 5. Conclusions

This preliminary study examined the longer term effects of Green Exercise on night-time HRV. The present study suggests an augmentation of night-time vagal activity following a short lunchtime walk in nature compared to a comparable Built walk. This suggests that a one-off bout of Green Exercise may have long lasting physiological effects. Further work is required in this area to explore the impact Green Exercise can have on restoration and recovery particularly during sleep and overnight ANS measures.

## Figures and Tables

**Table 1:** Overnight sleep duration and HRV measures following green or built walks.

Outcome Variable	Green Walk (*n* = 12)	Built Walk (*n* = 12)	Mean Difference (95% CI)	Main Effect (Condition)	Effect Size (Condition)	*Adj Main Effect	* Adj Effect Size
**Sleep Duration (min)**	425.0 (77.1)	445.0 (57.7)	−20 (−33 to −73)	*F* = 0.909 (*p* = 0.368)	*η_p_*^2^ = 0.102	*F* = 0.284 (*p* = 0.610)	*η_p_*^2^ = 0.039
**RR (ms)**	1018.2 (181.5)	1017.2 (189.7)	−0.9 (−73.0 to 71.2)	*F* = 0.001 (*p* = 0.976)	*η_p_*^2^ = 0.000	*F* = 0.043 (*p* = 0.840)	*η_p_*^2^ = 0.004
**SDNN (ms)**	43.6 (21.0)	37.0 (19.5)	6.6 (0.0 to 13.1)	*F* = 5.47 (*p* = 0.039)	*η_p_*^2^ = 0.332	*F* = 2.37 (*p* = 0.155)	*η_p_*^2^ = 0.192
**rMSSD (ms)**	47.4 (27.3)	38.8 (25.0)	8.6 (−0.1 to 17.3)	*F* = 5.36 (*p* = 0.041)	*η_p_*^2^ = 0.328	*F* = 3.40 (*p* = 0.095)	*η_p_*^2^ = 0.254
**SD1 (ms)**	33.8 (19.4)	27.7 (17.8)	6.1 (0.0 to 12.3)	*F* = 5.36 (*p* = 0.041)	*η_p_*^2^ = 0.328	*F* = 3.35 (*p* = 0.097)	*η_p_*^2^ = 0.251

Values and mean difference are the unadjusted means (SD). Main effect (condition) from repeated measures ANOVA. * Adjusted main effect from repeated measures ANOVA controlling for order. Mean Difference (95% CI) are derived from estimated marginal means. SDNN-Standard deviation of RR interval; rMSSD -root mean squared of successive differences and SD1—SD from Poincare plot.

**Table 2:** Differences in heart rate and perceived exertion during exercise following green or built walks.

Outcome Variable	Green	Built	Mean Difference (95% CI)	Effect (Condition)
HR (bpm)	107.5 (12.7)	109.5 (11.5)	−2.00 (−0.58 to 4.62)	*p* = 0.115
HR above rest (∆ bpm)	39.7 (14.1)	39.2 (13.8)	0.521 (−2.67 to 3.71)	*p* = 0.726
RPE	10.0 (1.6)	10.6 (1.8)	−0.636 (−1.50 to 0.228)	*p* = 0.132

Values and mean difference are the unadjusted means (SD). Main effect (condition) from repeated measures *t*-test. HR—Heart Rate; RPE—Ratings of perceived exertion.
